# Treatment with pirfenidone for two years decreases fibrosis, cytokine levels and enhances CB2 gene expression in patients with chronic hepatitis C

**DOI:** 10.1186/1471-230X-14-131

**Published:** 2014-07-27

**Authors:** Lucia Flores-Contreras, Ana S Sandoval-Rodríguez, Mayra G Mena-Enriquez, Silvia Lucano-Landeros, Inmaculada Arellano-Olivera, Arnulfo Álvarez-Álvarez, M Guadalupe Sanchez-Parada, Juan Armendáriz-Borunda

**Affiliations:** 1 Departamento de Biología Molecular y Genómica, Instituto de Biología Molecular en Medicina y Terapia Génica, CUCS, Universidad de Guadalajara, Sierra Mojada # 950, Guadalajara, Jalisco 44281, Mexico; 2 Unidad Médica de Alta Especialidad, Hospital de Especialidades Centro Medico Nacional de Occidente, Guadalajara, Jalisco, Mexico; 3 INNOVARE, Guadalajara, Jalisco, Mexico

**Keywords:** Pirfenidone, Liver fibrosis, Necroinflammation, Cytokines, Cannabinoid receptor 1, Cannabinoid receptor 2, Chronic hepatitis C

## Abstract

**Background:**

The aim of this study was to assess whether two-years treatment with Pirfenidone influences necroinflammation, fibrosis and steatosis, serum levels of TGF-β1, IL-6, TNF-α and CB1 and CB2 gene expression, in patients with chronic hepatitis C (CHC).

**Methods:**

Twenty-eight patients out of 34 with CHC virus infection were enrolled in the study and received Pirfenidone (1200 mg/day) for 24 months. Six patients dropped out after 12 months of PFD. Liver biopsies and serum samples were obtained at the beginning and end of treatment. Modified HAI was calculated. CB1 and CB2 gene expression was correlated with fibrosis progression alongside with necroinflammation and steatosis. TGF-β1, IL-6, TNF-α and liver transaminases were measured in serum at two-months intervals. HCV genotype and viral load were also assessed. Quality of life was evaluated by SF36 questionnaires and the prognosis of disease was assessed with Child-Pugh score. The Wilcoxon test matched-pair signed ranks were used to analyze the outcomes.

**Results:**

Intention to treat analyses were performed for biochemistry and clinical parameters. At the end of treatment, necroinflammation grading was reduced in an average of 3.2 points in 82% of patients (p < 0.05) and Ishak’s fibrosis stage decreased 2-points average in 67% of patients (p < 0.05). Steatosis decreased in 61% of patients. IL-6 and TGF-β1 serum levels decreased significantly in 93% and 67% of patients (p < 0.05), respectively, while TNF-α diminished in 47% of patients. ALT and AST tended to normalize in 81% of patients; CB2 mRNA levels increased in 86% and CB1 expression diminished in 29% of patients. Both, quality of life and Child-Pugh score improvements were reported in all patients.

**Conclusions:**

Pirfenidone for two years benefits CHC patients and improves inflammation, fibrosis and steatosis in higher number of patients as previously shown for 12-months treatment with PFD. Additionally, PFD improved TGFβ1 and IL-6 levels and diminished liver expression of anti-fibrogenic receptor CB2.

**Trial registration:**

ClinicalTrials.gov identifier: NCT02161952. Protocol Registration Date: 06/11/2014.

## Background

Chronic hepatitis C (CHC) is one of the most common etiologies for liver fibrosis and will eventually progress to cirrhosis or even to hepatocellular carcinoma [1,2]. Nowadays, it is estimated that up to 3% of the world population is affected by CHC, thus, a great deal of drugs designed to clear the liver from the infectious viral component in this disease, have been developed [2-6]. Nevertheless, fibrotic sequels eventually leading to dysfunctional liver activity in these patients are far from being resolved. In this frame of mind, pirfenidone (PFD) (5 methyl-1-phenil-2 (1H)-pyridone) has proved anti-fibrotic and anti-inflammatory properties in a wide number of animal models of fibrosis. PFD effects are mediated in part through inhibition of NF-κ-B activation, these mechanisms included inhibition of PDGF, hepatic stellate cells (HSC) proliferation, reduction of TNF-α and IFN-α levels and decrease in iNOS/NO induction [7,8]_._ Also, PFD down-regulates TGF-β1, TIMP-1, MMP-2 mRNA and collagen deposition [9,10]_._ Previously, our group demonstrated that one-year treatment with 1200 mg/day of oral PFD to patients with established liver fibrosis, decreased liver necroinflammation, steatosis and at less extent, fibrosis. Collagen I, TGF-β1 and TIMP-1 mRNAs were also down-regulated [11]_._ Among the many factors known to influence hepatic fibrosis progression (gender, age at HCV-infection, alcohol consumption), cannabinoids consumption has also been studied [12-14]. Recently, Zampino and cols. have highlighted that HCV-related clinical conditions like fibrosis, cirrhosis and hepatocellular carcinoma are the result of liver and systemic chronic inflammation [15]. On the other hand, cannabinoids signal through a G protein-coupled receptors called CB1 and CB2 which are absent or expressed in low levels in non injured livers, but strongly up-regulated in fibrotic-liver, especially in hepatic myofibroblasts and vascular endothelial cells [16-18]. Several studies have shown that endogenous and exogenous cannabinoid ligands and their receptors play a key role in the pathogenesis of chronic liver injury [19-21]. Based in our previous report [11], we aimed in this work to implement a 24 months clinical protocol with 1200 mg/day of oral PFD to analyze its effect in CB1 and CB2 cannabinoid receptors expression, serum levels of IL-6, TNF-α, TGF-β1 and necroinflammation and fibrosis scores. We reasoned that fibrosis stage in liver tissue from patients with CHC reported in this communication deserved further consideration, since an extended period with PFD treatment could result in an enhanced resolution of fibrosis as determined by liver biopsy. Furthermore, a couple of interesting articles recently published on the use of PFD in the treatment of two fibrotic diseases as diabetic nephropathy and pulmonary fibrosis suggest that PFD improves organ functionality demonstrating a benefit when it is used to treat fibrotic human pathologies [22,23]. Specifically, this very interesting study entitled “A Phase 3 Trial of Pirfenidone in Patients with Idiopathic Pulmonary Fibrosis” deserves a further and careful consideration [23].

## Methods

### Patients

Consecutive patients seen in our department were enrolled if they met the following criteria: (1) patients with established advanced liver disease caused by hepatitis C virus chronic infection defined by a positive test for anti-HCV antibodies and detectable serum HCV RNA (Amplicor HCV 2.0 PCR test system; Quest Diagnostic, San Juan Capistrano, CA, USA) (2) signing of an informed consent form to allow collection of liver biopsies before and after (3) no anti-fibrotic, antiviral or immunosuppressive drugs for at least 6 months before starting pirfenidone therapy; and (4) no alcohol intake and non-smokers of cannabis for at least 6 months before nor during PFD treatment. The baseline period was defined as an observational period before pirfenidone therapy and each patient was used as its own control.

The no-inclusion criteria were the following: (1) evidences for other forms of liver diseases (2) co-infection with hepatitis B virus or HIV; (3) post-transplant patients; (4) known intolerance to pirfenidone; (5) pregnancy or breast-feeding; (6) gastrointestinal bleeding; (7) malignancy; and (8) patients with concomitant disease such as heart failure, coronary artery disease, diabetes or cancer.

### Study design

This is an open-label, non-controlled and non-randomized clinical trial, designed to be carried out for 24 months in patients with chronic hepatitis C (CHC). The trial site was Institute of Molecular Biology in Medicine and Gene Therapy, University of Guadalajara, Mexico.

In this study 34 patients older than 18 years of age were enrolled, which had not previously participated in any other clinical protocol. Enrolled patients provided a medical history; complete physical examination was performed and intravenous blood was collected to assess liver function, liver fibrosis markers, complete blood count, blood coagulation profile, urinalysis, electrolytes status and serology (HCV, HBV, and HIV). HCV genotype was determined by LIPA (Quest Diagnostic, San Juan Capistrano, CA, USA) and HCV viral load was measured by PCR Quant (Amplicor HCV 2.0 PCR test system of Quest Diagnostic, San Juan Capistrano, CA, USA). Results were expressed as log10 IU/mL. Abdominal ultrasonography with Doppler assessment and computed tomography scan were performed to assess ascites and liver disease and portal system. Child-Pugh score was used to evaluate the severity of liver disease in all patients. Age at HCV infection, source of contamination and body mass index (BMI) were determined. Duration of HCV infection was estimated as the difference between the date of infection and the date of baseline liver biopsy. Two liver biopsies were obtained; one at baseline and a second one after two years of treatment.

Pirfenidone was administered three times a day in the form of 400 mg capsules manufactured according to standard good manufacturing practices (GMPs); good laboratory practices (GLPs) and sanitary regulations enforced by the Federal Commission for Protection against Sanitary Risks (COFEPRIS).

During the study, patients had twelve medical visits during a 24 months period, in which patients were physically examined, adverse event story was collected and laboratory testing was realized. Patients also completed the 36 item Short-Form Health Survey (SF-36). Health survey was applied to evaluate quality of life in patients before and after PFD treatment with their own initial data as control.

This safety/efficacy, nonrandomized, self-monitored, open phase study was approved by Ethical Committee from Hospital Civil de Guadalajara with registration number 505/05 and the COFEPRIS (Mexican Ministry of Health) with registry number 05330020020126. Furthermore, the protocol was registered in ClinicalTrials.gov identifier: NCT02161952.

Also, this study was undertaken in accordance with the Declaration of Helsinki and with local laws and regulation applicable to the use of drugs in Mexico, and all patients gave informed consent.

### Medication

Pirfenidone was supplied orally in 400 mg gel capsules three times daily (every 8 hours) for a full dosage of 1200 mg daily. All patients were instructed to take pirfenidone 20 minutes after meals to minimize gastrointestinal symptoms during 24 months. Patient’s compliance of drug intake was assessed using specifically designed drug registration sheets.

### Biomarker analysis

Biomarkers were analyzed in plasma from samples collected at baseline visit and end of study visits. Blood biomarkers measured were IL-6, TNF-α and TGF-β1. Cytokine analysis was measured using a conventional automated analyzer (Sincron-Cx7 analyzer) and pre-coated ELISA assay (R & D Systems, Minneapolis, MN, USA). All ELISA kits were utilized as described by the manufacturers.

### Liver histopathology

Liver biopsy was obtained before starting treatment with pirfenidone and second liver biopsy after two years of treatment using Color Doppler Sonography Needle-Guided. Liver biopsy specimens were fixed in formalin, embedded in paraffin, and stained with hematoxylin-eosin (H&E) and trichromic Masson. Fibrosis and necroinflammation were analyzed according to the Modified histological activity index (HAI) of Ishak scoring system [24]. Hepatic steatosis was measured according to the percentage of fat vacuoles in 20 random fields using a computer-assisted automated image analyzer (Image Pro-Plus 5.0, Media Cybernetics, Inc, Bethesda, MD, USA). Necroinflammation was scored by the activity index on a scale of 0 to 18. Fibrosis was staged on a scale of 0 to 6 (F0 or F6, F6 defining cirrhosis) with fibrosis stage of 4 or 5 defined as severe or advanced fibrosis. The 50% of patients enrolled in this study displayed advanced liver fibrosis (stage 4-5); 30% of patients had cirrhosis (stage 6) at the beginning of the trial and the 20% of them displayed mild or moderate liver fibrosis (stage 1-3).

### RT-PCR analysis

A portion of liver biopsy was frozen for RT-PCR analysis. Total RNA from liver biopsy was extracted using Trizol reagent according to the manufacturer instructions (Invitrogen, Carlsbad, CA, USA). RNA was quantified using spectophotometry. Reverse transcription was performed with 2 μg of total RNA for all genes with the cDNA synthesis kit (Cat. 4368814). Using 240 ng of random primer, 2U RNAse inhibitor, 5 mM of DTT, 1 mM of dNTPs and 200U of transcriptase, PCR was performed using the following protocol: 65°C/5 min, 4°C/5 min, 25°C/5 min, 50°C/60 min, 70°C/15 min and 4°C/5 min.

Quantitative real time PCR was performed using a Rotor Gene 3000 Termocycler (Corbett Research, Cambridge shire, UK) under the following conditions: 1 hold for 2 min at 50°C,1 hold for 5 min at 94°C, and 45 cycles of 30 sec at 94°C and 40 sec at 60°C. Specific primers for CB1 and CB2 were acquired from Applied Biosystems, NJ, USA. GAPDH was used as housekeeping gene. For the reaction, 2 μl of cDNA was used in 5 μl of Mix, 0.5 μl of TaqMan probe and primers for CB1 (Cat#:Hs00275634_m1), CB2 (Cat#:Hs00361490_m1) and GAPDH (Cat#:Hs99999905_m1). Gene expression was calculated with the 2^-ΔCT^ method according to Livak *et al*[25].

### Statistical analysis

Because values in baseline and treatment periods did not follow a parametric distribution, the Wilcoxon matched-pair signed ranks test was used to analyze the outcomes. Intention to treat analysis (ITT) was also performed. Data are presented as mean ± SD for parametric data. Statistical analysis was performed using Prism software (GraphPad Prism, CA, USA) Significance was defined as a P value <0.05.

## Results

### Study group characteristics

34 patients enrolled with chronic hepatitis C tolerated fairly well a dose of 1200 mg/day of PFD. During this latter period, 6 of 34 patients (17% drop-outs) were excluded from study for non-compliance or death (Table 1). These patients attended the protocol a range of 13 to 18 months. Therefore, the intention to treat analysis includes all of the 34 patients with CHC, although only 28 patients ended treatment for 24 months. None of the patients dropped out from the study due to severe side effects of PFD (Table 2). Mean age for patients was 56 ± 10 years and, as reported in other studies, females seemed more susceptible to be chronically infected with HCV (62%) [26]. Median body mass index was 28 ± 7; while 36% of patients presented overweight and 43% were obese according to WHO criteria [27]. Median age at HCV exposure was 27 years old and 32 years was the mean infection period. Predominant HCV transmission route was blood transfusion since 30 out of 34 patients underwent one before 1989. Main HCV genotype was 1a y 1b (70%), followed by genotypes 2a y 2c (12%), and 3a (6%). Serum HCV viral load remained without significant changes throughout the study, since values did not modify more than one log, indicating no noticeable effect of PFD treatment in HCV replication. Patient characteristics are summarized in Table 3.

**Table 1:** Causes of Death in patients enrolled in the study

	**Number of patients**	
**Duration of treatment**	**< 1 year**	**>1 year**
Treatment-related mortality	-	-
Hepatocarcinoma	-	1
Hepato-renal syndrome	-	1
Esophagic varices bleeding	-	1
Heart attack	-	1
Percent of enrolled patients death	0%	9%

Note: 2 patients were excluded from the study due to lack of monitoring.

**Table 2:** Secondary effects associated with pirfenidone treatment for two years

**Symptoms**	*n = **28**	**%**	**n = **34**	**%**
Gastritis	17	81%	23	67%
Nausea	10	48%	12	35%
Rash	10	48%	10	29%
Photosensitivity	3	14%	3	9%
Headache	3	14%	4	12%
Vomiting	1	5%	1	3%
Dizziness	2	10%	2	6%
Weakness	2	10%	2	6%
Insomnia	1	5%	1	3%
Somnolence	1	5%	1	3%

*Data in the 28 patients that conclude the study. Some patients presented more than one secondary effect.

**The most common side secondary effect in the 34 enrolled patients including the 6 drop-out patients were gastritis, nausea and rash, these effects disappeared after 3 months of treatment.

**Table 3:** General characteristics of patients

**Characteristics**		***n *** **= 34**
**Sex**	**Male, n (%)**	**13 (38%)**
	**Female, n (%)**	**21 (62%)**
Age at exposure (yr)	Mean ± SD	27 ± 9
Age at liver biopsy before treatment (yr)	Mean ± SD	56 ± 10
Age at liver biopsy after treatment (yr)	Mean ± SD	58 ± 10
Route of transmission	Blood transfusion, n (%)	29 (85%)
	Nosocomial, n (%)	5 (15%)
Duration of HCV exposure (yr)	Mean ± SD	32 ± 10
HCV genotype	Genotype 1, n (%)	24 (70%)&
	Genotype 2, n (%)	4 (12%)&
	Genotype 3, n (%)	2 (6%)
	ND, n (%)	5 (15%)
Change in HCV viral load after treatment	Increase 1 log, n (%)	1 (4%)
	Unchanged^*^, n (%)	27 (96%)
Body mass index (Kg/m^2^)	Mean (±SD)	28 ± 7^**^

&A patient presented co-infection with two genotypes of VHC. ^*^Unchanged: modification less than 1 log in viral load; ^* *^Overweight according to OMS; ND: not determined. HCV: Hepatitis C virus.

### Characteristics of dropping-out patients

As previously stated, of the initial 34 patients enrolled, six patients stopped therapy after at least 1 year of therapy due to non-compliance or death. Reason for death and non-compliance were not related to pirfenidone secondary effects. Deaths were due to advance liver disease complications like hepato-renal syndrome (1 patient, Child-Pugh C), hepatocarcinoma (1 patient, Child-Pugh B), esophagic varices bleeding (1 patient, Child-Pugh B) or non-related to liver disease like heart attack (1 patient, Child-Pugh A). Two patients stop attending the trial since they were from out state Jalisco (Child-Pugh A) (Table 4).

**Table 4:** Severity of disease according to child-pugh score

			**Child-pugh score**		
**Treatment**	**No. patients**	**Statistics**	**Before**	**After**	***P value***
< 2 years	6	Minimum	5 (A)	6 (A)	*0.2*
		Median	6.5 (A)	9 (B)	
		Maximum	10 (C)	9 (B)	
2 years	28	Minimum	5 (A)	5 (A)	*0.8*
		Median	5.5 (A)	6 (A)	
		Maximum	10 (C)	12 (C)	
Intention to treat	34	Minimum	5 (A)	5 (A)	*0.6*
		Median	6 (A)	6 (A)	
		Maximum	10 (C)	9 (B)	

### Effect of PFD on liver biochemistry and clinical data

AST and ALT and bilirubins in serum showed elevated values at enrollment suggesting active hepatic damage. All biochemical parameters tend to decrease after treatment. 81% of patients (28) who completed treatment for two years reduced enzyme values compared to their own initial data. Statistically significant reduction was achieved in AST (94 ± 55 *versus* 75 ± 38 UI/mL; p < 0.05) while ALT reduced from 85 ± 71 to 65 ± 34 UI/mL; the levels of total bilirubin diminish from 1 ± 0.8 to 1 ± 0.5 and indirect bilirubin 0.8 ± 0.7 to 0.6 ± 0.4 between initial mean values and after treatment mean values of all patients (Table 5).

**Table 5:** Biochemical measurements of patients

**Treat**	**< 2 years**			**2 years**			**Intention to treat**		
**No. patients**	**6**			**28**			**34**		
**Biochemical data**	**Before**	**After**	***P value***	**Before**	**After**	***P value***	**Before**	**After**	***P value***
ALT (mean ± SD)	67 ± 25	59 ± 20	*0.63*	85 ± 71	65 ± 34	*0.13*	82 ± 68	64 ± 32	*0.23*
AST (mean ± SD)	95 ± 38	87 ± 38	*1.0*	94 ± 55	75 ± 38	0.02*	94 ± 54	78 ± 38	0.02*
Total Bilirubin (mean ± SD)	2 ± 1.7	3.1 ± 1.5	*0.16*	1 ± 0.8	1 ± 0.5	0.04*	1.4 ± 0.9	1.4 ± 1.1	*0.46*
Direct Bilirubin (mean ± SD)	0.8 ± 0.5	1.5 ± 0.9	0.03*	0.4 ± 0.3	0.4 ± 0.2	*0.63*	0.5 ± 0.3	0.6 ± 0.6	*0.46*
Indirect Bilirubin (mean ± SD)	1.3 ± 0.9	1.6 ± 0.9	*0.56*	0.8 ± 0.7	0.6 ± 0.4	0.05*	0.9 ± 0.7	0.8 ± 0.6	*0.31*

ALT: Alanine transaminase; AST: Aspartate transaminase. *p< 0.05.

### Child-Pugh score in enrolled patients

Child-Pugh score was measured in order to indirectly assess the prognosis of chronic liver disease and to correlate with patient survival at two years [28]. The 28 patients that concluded the two-year treatment showed an improved score at the end of the study (Table 4). Hepatitis C patients with established liver fibrosis after PFD treatment had improved Child-Pugh score compared to initial values as seen in Table 4. Treatment allowed patients to remain in a compensated status, since 20 out of 28 patients experienced no change in Child-Pugh A or B score, 5/28 improved their score and only 3 patients decreased Child-Pugh score indicating most severe liver damage. However, when drop-out patients were included, no statistical significance was achieved in this score.

### Intention to treat analysis for quality of life

SF-36 is one of the questionnaires most recognized to monitor quality of life. The intention to treat analysis for the quality of life showed scores in SF-36 significantly higher in all enrolled patients compared at the end and baseline of treatment, demonstrating that quality of life improved in 97% of them as described in Table 6. Data for the diverse domains of the questionnaire in the 34 patients enrolled were as follows: physical function 82 ± 23, physical role 86 ± 31, body pain 82 ± 19, general health 73 ± 18, vitality 73 ± 22, social role 81 ± 23, emotional role 83 ± 38, and mental health 83 ± 17. All parameters improved at the end of the treatment.

**Table 6:** Intention-to-treat analysis for quality of life

**Domains (mean ± SD)**	SF-36* before	SF-36* after	***P value***
Physical function	69 ± 30	82 ± 23	0.00**
Role physical	61 ± 47	86 ± 32	0.02**
Body pain	70 ± 26	81 ± 19	0.00**
General health	55 ± 21	73 ± 18	0.00**
Vitality	62 ± 24	73 ± 22	0.02**
Social functioning	73 ± 29	81 ± 23	0.03**
Role emotional	68 ± 45	83 ± 38	*0.17*
Mental health	75 ± 20	83 ± 17	0.00**

*SF-36: Short-Form Health Survey with 36 questions. **p < 0,05.

### Secondary effects

Pirfenidone was well tolerated. All patients tolerated the dosage of 1200 mg/day from the beginning of the treatment; seven patients did not develop any secondary effect. Table 2 summarizes secondary effects known to be associated with pirfenidone for 27/34 patients that showed any or several of them. Patients developed negligible secondary effects, like gastritis (23/34 patients; 64%%), nausea (12/3 patients; 35%) among others. These secondary effects disappeared 3 months after initiating PFD intake. None of patients dropped out of the study due to side effects of treatment. All patients that conclude the study adhered to treatment (they received ≥80% of scheduled doses).

### PFD effect in liver histopathology

**Figure 1:**
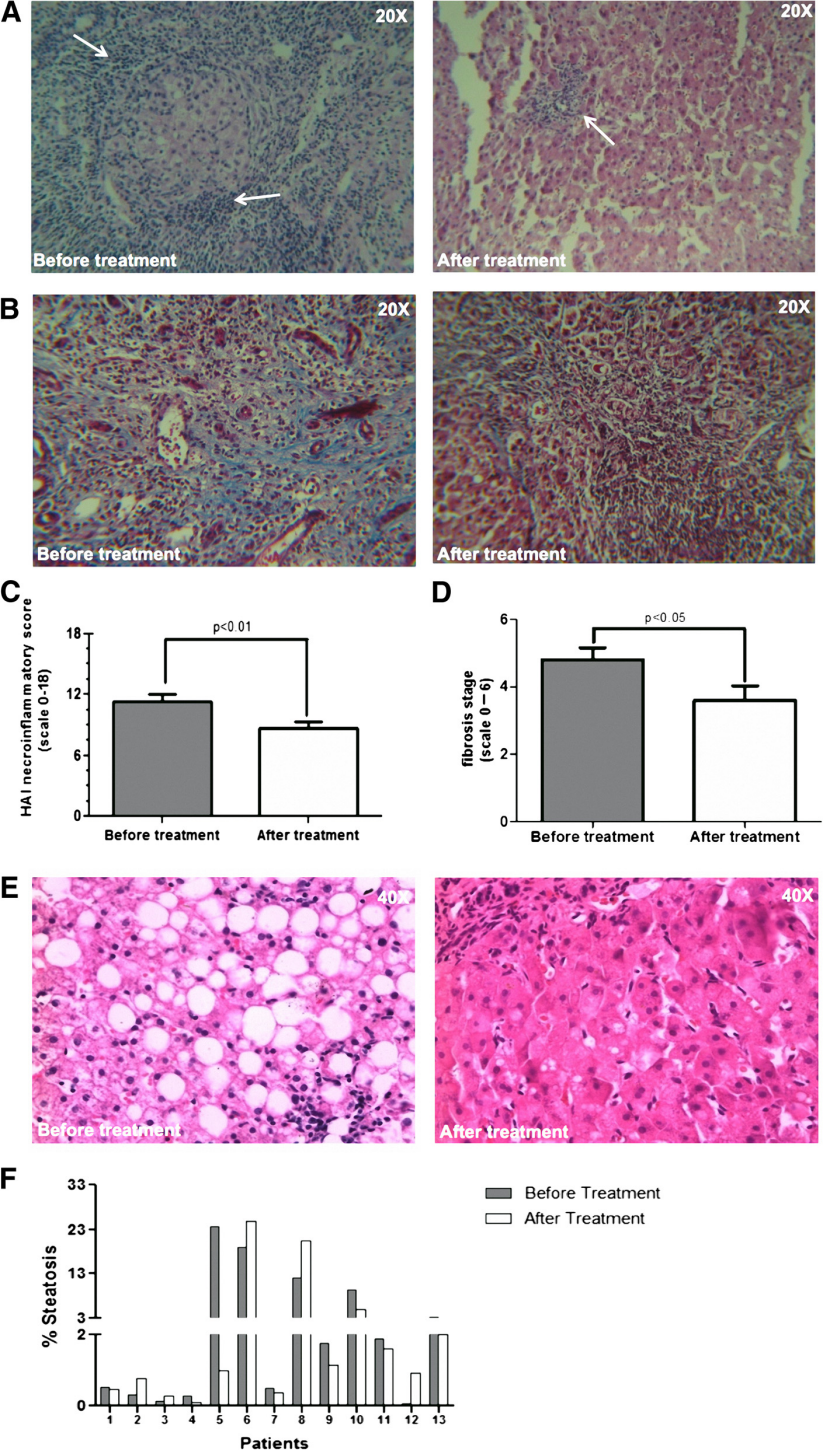
**Pirfenidone treatment decreases necroinflammation grade, fibrosis stage and steatosis.** Representative photomicrographs of liver biopsy were stained with H&E showing necroinflammation **(A)** and trichromic Masson staining for fibrosis **(B)** before and after of treatment. In **(A)** arrows indicate that inflammatory cell infiltrate is reduced post-treatment. In **(B)** collagen fibers clearly are reduced after PDF treatment. **(C)** Graph indicates mean ± SD initial and final values for necroinflammation grade that reduced an average of 2.6 points in 81.8% of patients (p < 0.01). Mean ± SD initial and post-treatment fibrosis stage is represented in **(D)**; stage decreased 2 points as average in 67% of patients (p < 0.05) by the end of treatment. **(E)** Representative sections of liver tissue were stained with H&E to determine steatosis area. Representative microphotographs (patient 05) showed before treatment macro-steatosis and micro-steatosis, and an obvious decline in steatosis is evident by the end of treatment. **(F)** Individual data for percentage of steatosis indicates decrease in liver fat-area in 8/13 patients, while in 5/13 patients steatosis remains.

At the end of 24 months of treatment, necroinflammation score was reduced an average of 3.2 points in 82% of patients (p < 0.01) and fibrosis decreased in 67% of patients by 2-point average according to Ishak’s staging. Representative photographs of liver biopsy from one patient before and after treatment are shown in Figure 1. Initially, hepatic fibrosis with regenerative nodules and necroinflammation was observed, while after two-year PFD treatment H&E staining indicates that necroinflammation decreases since inflammatory cells infiltration is reduced along with an improvement in tissue morphology (Figure 1A). Necroinflammation grading graph shows reduction in the mean value obtained from all patients after PFD treatment compared to initial data (Figure 1C). Before treatment, patient necroinflammation grading ranged between 8 and 16 and after PFD treatment, classifications reduced to 6-11 points. Liver fibrosis was markedly attenuated after PFD treatment in 67% of patients compared to their own initial scores (Figure 1B). In these patients, quantitative analysis of fibrosis demonstrated a significant reduction from 4.8 ± 1.1 to 2.8 ± 0.7 points in the median fibrosis Ishak stage (p < 0.05). Patients with reduced fibrosis showed mild fibrosis after treatment as observed in trichromic Masson staining that displayed a noticeable diminution in fibrotic septums where extracellular matrix is reduced in thickness (Figure 1D). Besides, steatosis was detected in thirteen patients. Steatosis has been associated with progression of liver fibrosis. Figure 1E shows representative images of steatosis improvement achieved in representative patient number eleven. Steatosis was quantitatively measured showing a significant reduction in most patients evaluated. In these patients, a significant reduction in steatosis was achieved (p < 0.01) from 4.9% to 1.24% after treatment. In Figure 1F steatosis data for each patient is presented as percentage of total stained area. Previously, we reported that one-year treatment with PFD rendered reduced necroinflammation and fibrosis, as well as, steatosis in patients with advanced established liver fibrosis. Now, in this study we demonstrated that two year-treatment with PFD is more effective achieving a major decrease in necroinflammation, fibrosis and steatosis.

### Effects of PFD on biomarkers

**Figure 2:**
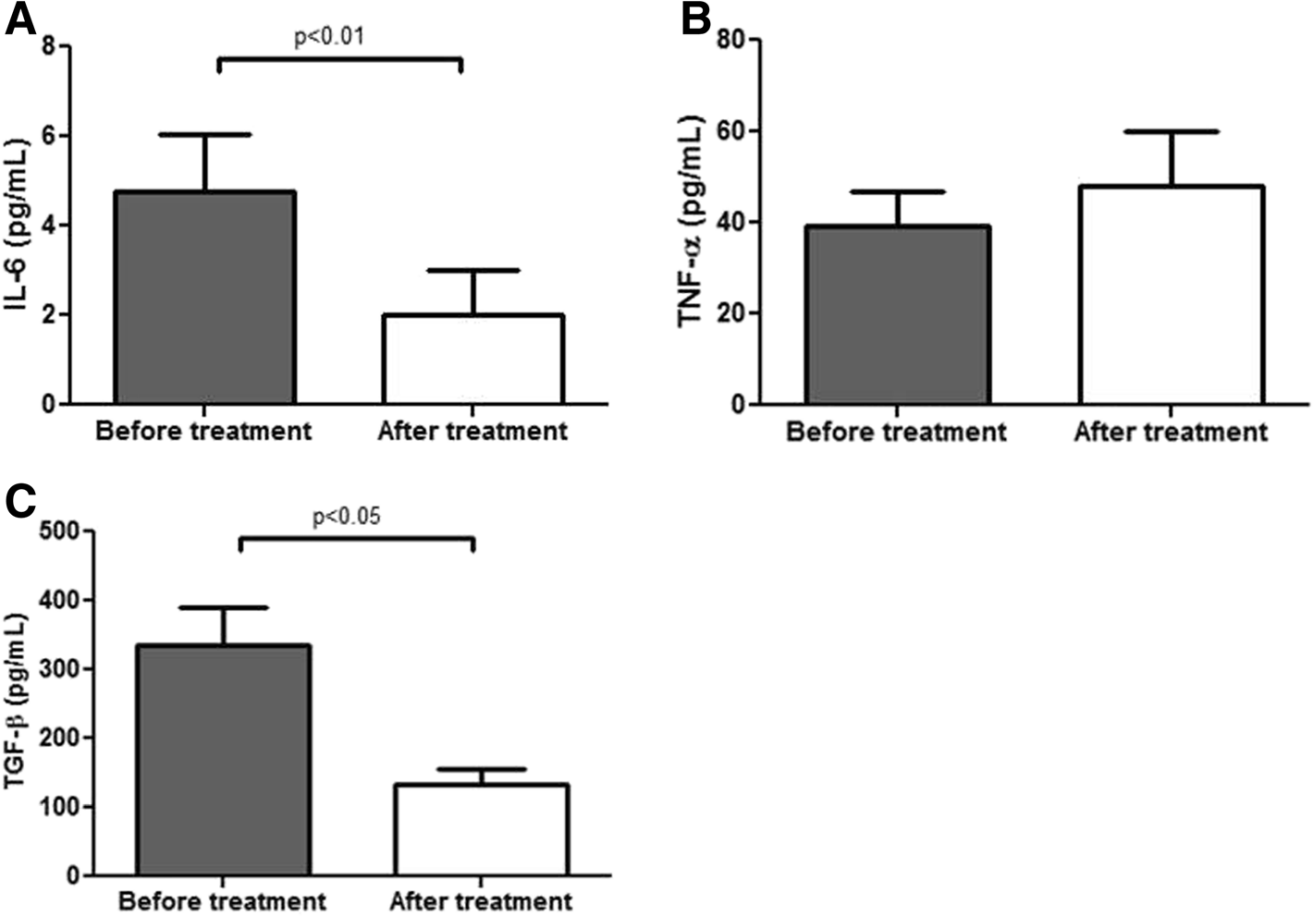
**TGF-β1 and cytokines serum levels in CHC patients before and after treatment with PFD.** Quantitative analysis of cytokines was determined before and after PFD treatment. **(A)** After treatment IL-6 mean ± SEM decreased reaching statistical significance (p < 0.05). In **(B)** graph indicates TNF-α mean ± SEM in patients before and after treatment. **(C)** TGF-β1 mean ± SEM concentration of all patients after treatment (p < 0.05).

In order to correlate our histologic results with serum markers, pro-inflammatory cytokines IL-6 and TNF-α were analyzed. Figure 2A shows that IL-6 serum levels decreased significantly in 93% of patients from 4.7 ± 4.9 pg/mL before treatment to 2.0 ± 3.9 pg/mL after treatment (p < 0.01). In Figure 2B TNF-α serum levels are shown. Unexpectedly, we did not find changes after PFD treatment. This might be due to the sensitivity of the methodology used. Experiments are undergoing to overrule this possibility. TGF-β1, the emblematic pro-fibrogenic cytokine showed a trend to diminish in 67% of patients as seen in Figure 2C. TGF-β1 values were down regulated from 501.9 ± 442.4 pg/mL before treatment to 204.5 ± 174.2 pg/mL after treatment showing statistical significance against initial levels (p < 0.05).

### PFD effect on CB1 and CB2 receptors gene expression

**Figure 3:**
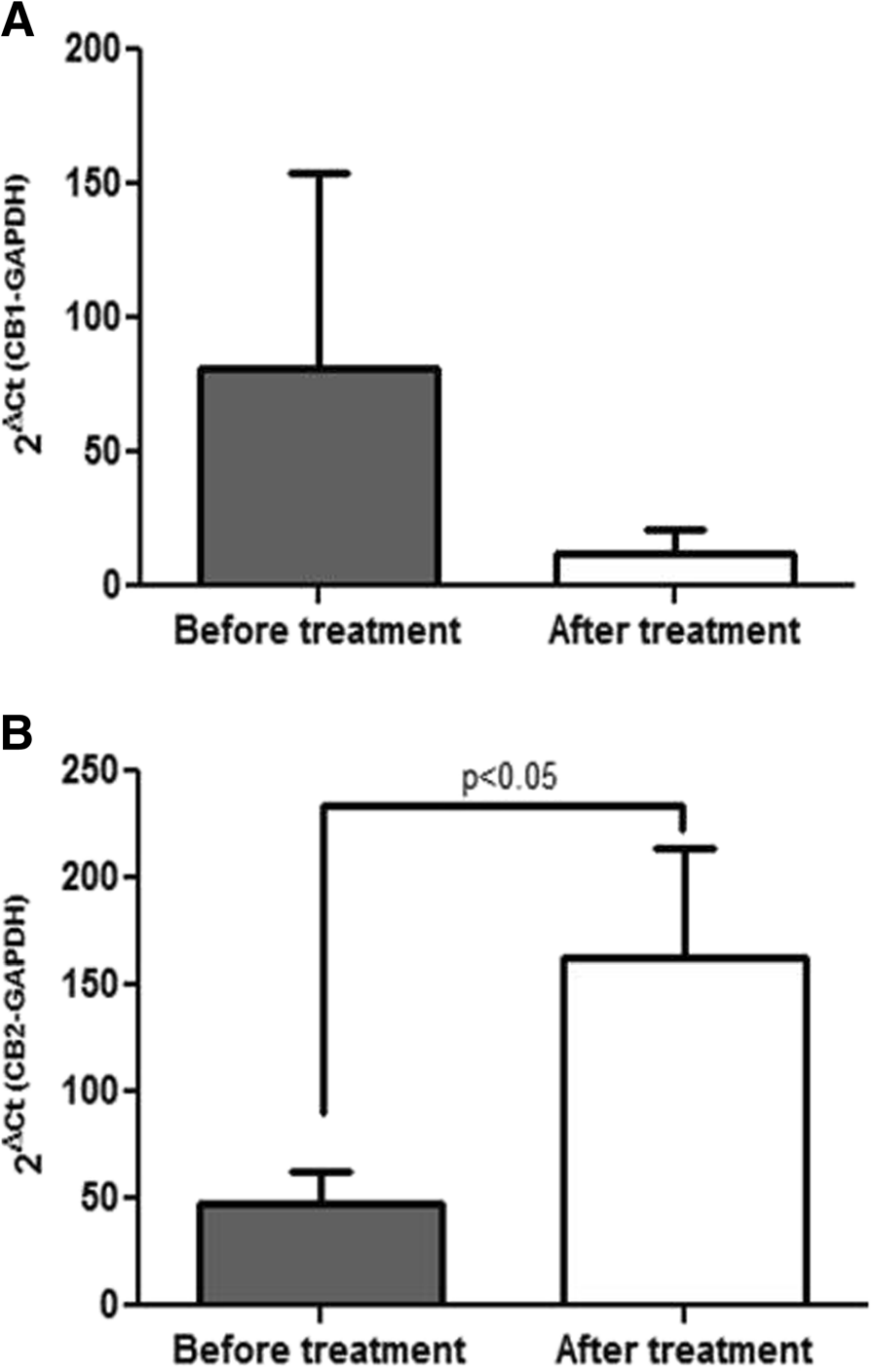
**Gene expression of CB-1 and CB-2 in CHC patients.** Messenger RNA levels of CB1 and CB2 receptors were analyzed by Real Time-PCR. In **(A)** mean ± SD values of all patients analyzed for CB1 receptor are presented with no statistical change, in **(B)** a decrease after treatment was evident, CB2 receptor mRNA was significantly up-regulated (p < 0.05) in patients after treatment.

Real time PCR was used to detect gene expression of key molecules involved in fibrosis progression. CB1 and CB2 dynamically participate in liver fibrosis, CB1 is considered pro-fibrogenic and its blockade has shown fibrosis reduction, while inducing CB2 signaling leads to anti-fibrogenic effects [19,29]. We detected cannabinoid receptors CB2 and CB1 mRNA levels in liver samples in order to analyze any possible effect of PFD treatment over these molecules. Expression of cannabinoid receptors CB1 and CB2 was detected in all patients analyzed. CB1 gene expression demonstrated a tendency to diminish at the end of treatment in 28.5% of patients with 4.7-fold decrease (Figure 3A). Although the statistic test used did not allow us to conclude significance on this issue, tendency of CB1 to decrease is clear. On the other hand, CB2 receptor gene expression augmented a 5.4 fold-increment in 85.7% of patients after treatment (Figure 3B). In these patients, statistical significance reached a p < 0.05 compared to initial values.

## Discussion

Our group has demonstrated that PFD is an anti-inflammatory and anti-fibrogenic agent in experimental models of cirrhosis [9] and in humans affected with hepatic fibrosis caused by different etiologies [11,30-32]_._ Moreover, PFD has shown to improve necroinflammation, steatosis and liver regeneration in a sub-set of patients with established advanced liver fibrosis caused by HCV infection [11]. It had also been demonstrated that PFD has an anti-fibrogenic action inhibiting capsular contracture in mammary implants and an antioxidant role in different experimental models of cirrhosis [31-33]_._ Moreover, two randomized, double-blind, multicentre studies recently published; used PFD in the treatment of pulmonary fibrosis and diabetic nephropathy, pointing out the relevance of PFD in the clinical scenario when it comes to treat patients with these fibrotic illnesses [22,23]. Moreover, in the present study we evaluated the effect of 24 months treatment with 1200 mg/kg of PFD in HCV chronically-infected patients to elucidate whether PFD therapy has an effect on serum expression of fibrogenic and pro-inflammatory molecules as well as other markers. At the molecular level, PFD possess a potent anti-TNF-α and anti-TGF-β1 action and on other pro-inflammatory cytokines [34-36]. It has been confirmed in this two-years follow-up trial that IL-6 experienced a significant decrease in serum levels in most of patients as well. This fact indicates that the inflammatory pathway IL-6/TNF-α is being affected by PFD treatment. According to this, necroinflammation score also reduces in 82% of patients after treatment; a number that is significant greater than the 53.3% achieved with one-year treatment. Interestingly, we showed that 2-year PFD treatment compared to 1-year treatment augments the number of patients that achieved fibrosis reduction (30% in the one-year follow up *vs*. 67% in the two-year study). This improvement in liver fibrosis was histologically monitored using Ishak score, and a considerably 2-point decrease (mean value) in liver fibrosis score was achieved at the end of treatment compared with patients-initial data. This information correlates with observed reduction on serum levels of hepatic enzymes and bilirubin that reflects a functional restoration in the liver due to PFD treatment.

As known, steatosis is the result of the accumulation of fat in hepatocytes and it has been associated with rapid progression of liver fibrosis [37,38]. The percentage of patients that reduced fat-occupied liver area with 2-year treatment was roughly the same as those in the one-year period (60% *vs* 61.5%). It is important to keep in mind, that studies have reported that agonist of CB1 receptor promotes steatosis and strong evidence argues for a steatogenic role of the cannabinoid system [14,39-41]. Therefore, histological findings in steatosis reduction can be related with diminution in CB1-mRNA levels observed in this study. In this framework, the endocannabinoid system plays an important role in liver fibrosis. In murine models of chronic liver-injury, CB1 receptor antagonism by pharmacological or genetic mechanisms reduced fibrotic area, TGF-β1 expression and accumulation of fibrogenic cells [29]. In opposition to CB1 receptor antagonism effects, CB2 receptor agonist counteracts liver fibrosis and induces inhibition and apoptosis of hepatic myofibroblasts and stellate cells [19]. Thus, we looked at the effects of PFD therapy in the expression of these molecules. To our knowledge, this report is the first attempt to search for a possible relation between PFD and CB1 and CB2 mRNA expression. After treatment, patients showed CB1 mRNA reduced to almost half of the initial level (even when no statistical difference was obtained) demonstrating benefits of treatment associated with steatosis decline; while CB2 mRNA levels were over-expressed approximately by 50% which correlates with the improvement in fibrotic score and necroinflammation. In this context, results from Coppola et al. related to rs35761398 variant of CB2 receptor gene (CNR2) in Italian HCV-chronic infected patients, demonstrated that QQ allele is associated with more severe inflammation and hepatocellular necrosis. The influence of this polymorphism in the response to pirfenidone in Hispanic patients could be evaluated in future studies [42].

As reported, CBs liver expression can be detected mainly in hepatocytes and stellate cells [19,29,43]. In this protocol, CBs mRNAs were detected in liver homogenates. Thus, we believe this reflects the organ microenvironment that induces liver improvement. As observed, PFD was satisfactorily tolerated for the two-years period at 1200 mg/day, given that 7/28 patients did not develop any secondary effect and 21 patients developed just negligible side effects like gastritis, nausea and rash. Several limitations of the study must be recognized, though our findings strongly support that PFD reduces liver fibrosis, necroinflammation and steatosis. Also, treatment for a period of two years is well-tolerated and increasing time of treatment renders enhanced benefits as observed in this HCV-liver fibrosis patient cohort, i.e. recovering in hepatic markers, TGF-β1 and pro-inflammatory cytokines serum levels reduction as well as, mRNA levels of CB2 mRNA increase.

## Conclusion

In conclusion, there is an evident advantage of two-years treatment over the one-year period and administration of PFD induced only minor side effects, which were resolved after 2/3 months of PFD intake. In addition, histopathologic results showed improvement in terms of the progression of fibrosis and stage of inflammation, as well as decrease in the percentage of steatosis after two years of treatment with PFD. Also, this study is the first to show that PFD decreases serum levels of TGF-β1 and IL-6 and gene expression increases anti-fibrogenic CB2 receptor. However, it is important to remember that viral clearance is indispensable to cure the disease and to resolve liver damage. In this context, direct antiviral agents (DAA) and immune system boosters (some of them with proven efficacy) are the standard of care for chronic HCV infected patients. However, these treatments are not available for general population who is not covered under the social services (i.e. regular health care in North-America) in developing countries like Mexico. This is due to the elevated cost of such a treatments, which make them inaccessible to non-affiliated patients. Then, in this majority of untreated patients with DAA, an alternative anti-fibrogenic therapy could impact their health and quality of life. Besides, the combination of pirfenidone with DAAs can be useful in patients infected with genotypes that can be hardly eliminated with standard therapies and can also be evaluated in other liver diseases.

## Abbreviations

ALT: Alanine transaminase; AST: Aspartate transaminase; BMI: Body mass index; CB1: Cannabinoid receptor 1; CB2: Cannabinoid receptor 2; CHC: Chronic Hepatitis C; GAPDH: Glyceraldehyde-3-phosphate dehydrogenase; HAI: Histological activity index; HCV: Hepatitis C virus; HIV: Human immunodeficiency virus; IL-6: Interleukin 6; INF-α: Interferon-alpha; iNOS: induced Oxide Nitric Synthetase; MMP-2: Matrix metalloproteinase-2; NF-κB: Nuclear factor kappa-light-chain-enhancer of activated B cells; NO: Nitric oxide; PFD: Pirfenidone; PDGF: Platelet-derived growth factor; RT-PCR: Real time- Polymerase chain reaction; TIMP-1: Tissue inhibitor of metalloproteinases-1; TGF-β1: Tumor growth factor- beta1; TNF-α: Tumor necrosis factor-alpha.

## Competing interests

Authors do not have anything to disclose regarding competing interest for this manuscript.

## Authors' contributions

LFC: Principal author, article writing, sample processing, laboratory studies, analysis of results and statistics. ASR: Methodological analysis and article writing. MME: Laboratory tests and analysis of results. SLL: Protocol administration and clinical evaluation. IAO: Patient recruitment and clinical evaluation. AAA: Pathological analysis in liver biopsies. MGSP: Laboratory tests. JAB: Principal investigator, liver biopsies analysis, article writing and supervision. All authors read and approved the final manuscript.

## Pre-publication history

The pre-publication history for this paper can be accessed here:

http://www.biomedcentral.com/1471-230X/14/131/prepub

## References

[B1] Friedman SL Liver fibrosis: from bench to bedside J Hepatol 2003 38 38 53 10.1016/s0168-8278(02)00429-4 12591185

[B2] Alter MJ Epidemiology of hepatitis C virus infection World J Gastroenterol 2007 7:13 17 2436 2441 17552026 10.3748/wjg.v13.i17.2436 PMC4146761

[B3] Liang TJ Rehermann B Seeff LB Hoofnagle JH Pathogenesis, natural history, treatment, and prevention of hepatitis C Ann Intern Med 2000 132 296 305 10.7326/0003-4819-132-4-200002150-00008 10681285

[B4] Memom MI Memom MA Hepatitis C: an epidemiological review J Viral Hepat 2002 9 84 100 10.1046/j.1365-2893.2002.00329.x 11876790

[B5] Zeuzem S Andreone P Pol S Lawitz E Diago M Roberts S Focaccia R Younossi Z Foster GR Horban A Ferenci P Nevens F Müllhaupt B Pockros P Terg R Shouval D van Hoek B Weiland O Van Heeswijk R De Meyer S Luo D Boogaerts G Polo R Picchio G Beumont M Telaprevir for retreatment of HCV infection N Engl J Med 2011 364 25 2417 2428 10.1056/NEJMoa1013086 21696308

[B6] Foote BS Spooner LM Belliveau PP Boceprevir: a protease inhibitor for the treatment of chronic hepatitis C Ann Pharmacother 2011 45 9 1085 1093 10.1345/aph.1P744 21828346

[B7] Wang F Wen T Chen XY Wu H Protective effects of pirfenidone on D-galactosamine and lipopolysaccharide-induced acute hepatotoxixity in rats Inflamm Res 2008 57 183 188 10.1007/s00011-007-7153-8 18344059

[B8] Tsuchiya H Kaibori M Yanagida H Yokoigawa N Kwon AH Okuma T Kamiyama Y Pirfenidone prevents endotoxin-induced liver injury after partial hepatectomy in rats J Hepatol 2004 40 94 101 14672619 10.1016/j.jhep.2003.09.023

[B9] Garcia-Benavides L Hernandez I Sandoval A Salazar A Garcia J Vera J Grijalva G Muriel P Margolin S Armendariz-Borunda J Pirfenidone effectively reverses experimental liver fibrosis J Hepatol 2002 37 797 805 10.1016/S0168-8278(02)00272-6 12445421

[B10] Di Sario A Bendia E Macarri G Candelaresi C Taffetani S Marzioni M Omenetti A De Minicis S Trozzi L Benedetti A The anti-fibrotic effects of pirfenidone in rat liver fibrosis is mediated by downregulation of procollagen alpha 1(l), TIMP-1 and MMP2 DigLiverDis 2004 36 11 744 751 10.1016/j.dld.2004.05.012 15571005

[B11] Armendariz-Borunda J Islas-Carbajal MC Meza-Garcia E Rincon AR Lucano S Sandoval AS Salazar A Berumen J Covarrubias A Arechiga G Garcia L A pilot study in patients with established advanced liver fibrosis using pirfenidone Gut 2006 55 11 1663 1665 10.1136/gut.2006.107136 17047115 PMC1860119

[B12] McCaughan GW George J Fibrosis progression in chronic hepatitis C virus infection Gut 2004 53 318 321 10.1136/gut.2003.026393 14960506 PMC1773949

[B13] Ortiz V Berenguer M Rayon JM Carrasco D Berenguer J Contribution of obesity to hepatitis C-related fibrosis progression Am J Gastroenterol 2002 97 2408 2414 10.1111/j.1572-0241.2002.05995.x 12358265

[B14] Hézode S Roudot-Thoraval F Nguyen S Daily cannabis smoking as a risk factor for progression of fibrosis in chronic hepatitis C Hepatology 2005 42 63 71 15892090 10.1002/hep.20733

[B15] Zampino R Marrone A Restivo L Guerra B Sellitto A Rinaldi L Chronic HCV infection and inflammation: clinical impact on hepatic and extra-hepatic manifestations World J Hepatol 2013 5 10 528 540 24179612 10.4254/wjh.v5.i10.528 PMC3812455

[B16] Mallat A Lotersztajn S Endocannabinoids and Liver Disease I: Endocannabinoids and their receptors in the liver Am J Physiol Gastrointest Liver Physiol 2008 294 9 12 10.1152/ajpgi.00467.2007 17975129

[B17] Tam J Liu J Mukhopadhyay B Cinar R Godlewski G Kunos G Endocannabinoids in liver disease Hepatology 2011 53 346 355 10.1002/hep.24077 21254182 PMC3073545

[B18] Siegmund SV Schwabe RF Endocannabinoids and liver disease. II. Endocannabinoids in the pathogenesis and treatment of liver fibrosis Am J Physiol Gastrointest Liver Physiol 2008 294 2 357 362 10.1152/ajpgi.00456.2007 18006606

[B19] Julien B Grenard P Texeira-Clerc F van Nhieu JT Li L Karsak M Zimmer A Mallat A Lotersztajn S Antifibrogenic role of the cannabinoid receptor CB2 in the Liver Gastroenterology 2005 128 742 755 10.1053/j.gastro.2004.12.050 15765409

[B20] Parfieniuk A Flisiak R Role of cannabinoides in chronic liver diseases World J Gastroenterol 2008 28:14 40 6190 6114 10.3748/wjg.14.6109 PMC2761570 18985799

[B21] Muñoz-Luque J Ros J Fernández-Varo G Tugues S Morales-Ruiz M Alvarez CE Friedman SL Arroyo V Jimenez W Regression of fibrosis after chronic stimulation of cannabinoid CB2 receptor in cirrhotic rats J Pharmacol ExpTher 2008 324 2 475 483 10.1124/jpet.107.131896 PMC2887659 18029545

[B22] Sharma K Ix JH Mathew AV Cho M Pflueger A Dunn SR Francos B Sharma S Falkner B McGowan TA Donohue M Ramachandrarao S Xu R Fervenza FC Kopp JB Pirfenidone for diabetic nephropathy J Am Soc Nephrol 2011 22 6 1144 1151 10.1681/ASN.2010101049 21511828 PMC3103734

[B23] King Talmadge E Bradford Williamson Z Castro-Bernardini S Fagan Elizabeth A Glaspole I Glassberg Marilyn K Gorina E Hopkins Peter M Kardatzke D Lancaster L Lederer David J Nathan Steven D Pereira Carlos A Sahn Steven A Sussman R Swigris Jeffrey J Noble Paul W A phase 3 trial of pirfenidone in patients with idiopathic pulmonary fibrosis N Engl J Med 2014 370 2083 2092 10.1056/NEJMoa1402582 24836312

[B24] Ishak K Baptista A Bianchi L Callea F De Groote J Gudat F Grootes J Gudat F Denk H Desmet V Korb G MacSeweeni RNM Phillips MJ Portmannl BG Paulsen H Scheuer PJ Schmid M Thaler H Histological grading and staging of chronic hepatitis J Hepatol 1995 22 6 696 699 10.1016/0168-8278(95)80226-6 7560864

[B25] Livak KJ Schmittgen TD Analysis of relative gene expression data using real time quantitative PCR and the 2(-Delta Delta C (T)) Method Methods 2001 25 4 402 408 10.1006/meth.2001.1262 11846609

[B26] De Torres M Poynard T Risk factors for liver fibrosis progression in patients with chronic hepatitis C Ann Hepatol 2003 2 1 5 11 15094700

[B27] World Health Organization [http://www.who.int/mediacentre/factsheets/fs311/en/]

[B28] Kamath PS Wiesner RH Malinchoc M Kremers W Therneau TM Kosberg CL D’Amico G Dickson ER Kim WR A model to predict survival in patients with end-stage liver disease Hepatology 2001 33 464 10.1053/jhep.2001.22172 11172350

[B29] Texeira-Clerc F Julien B Grenard P Nhieu JT Deveaux V Li L Serriere-Lanneau V Ledent C Mallat A Loterstajn S CB1 cannabinoid receptor antagonism: a new strategy for the treatment of liver fibrosis Nat Med 2006 12 6 671 676 10.1038/nm1421 16715087

[B30] Macias-Barragan J Sandoval-Rodriguez A Navarro-Partida J Armendariz-Borunda J The multifaceted role of pirfenidone and its novel targets Fibrogenesis Tissue Repair 2010 1 3 16 10.1186/1755-1536-3-16 PMC2944211 20809935

[B31] Gancedo M Ruiz-Corro L Salazar-Montes A Rincon AR Armendáriz-Borunda J Pirfenidone in capsular contracture after mammary implantation Aesth Plast Surg 2008 32 32 40 10.1007/s00266-007-9051-4 17968613

[B32] Veras-Castillo ER Cardenas-Camarena L Lyra-Gonzalez I Muñoz-Valle JF Lucano-Landeros S Guerrero-Santos J Ganzalez-Ulloa B Mercado-Barajas JL Sanchez-Parada MG Azabache-Wenneceslao R Armendariz-Borunda J Controlled clínical trial with pirfenidone in the treatment of breast capsular contracture Ann Plast Surg 2011 In press 10.1097/SAP.0b013e31822284f4 21712700

[B33] Salazar-Montes A Ruiz-Corro L Lopez-Reyes A Castrejon-Gomez E Armendariz-Borunda J Potent antioxidant role of pirfenidone in experimental cirrhosis Eur J Pharmacol 2008 24 595 69 77 18652820 10.1016/j.ejphar.2008.06.110

[B34] Nakazato H Oku H Yamane S Tsuruta Y Suzuki R A novel anti-fibrotic agent pirfenidone suppresses tumor necrosis factor-alpha at the translational level Eur J Pharmacol 2002 20 1-3 177 185 12098600 10.1016/s0014-2999(02)01758-2

[B35] Lyer SN Gurujeyalakshmi G Giri SN Effects of pirfenidone on transforming growth factor-beta gene expression at the transcriptional level in bleomycin hamster model of lung fibrosis J Pharmacol Exp Ther 1999 291 1 367 373 10490926

[B36] Cain WC Stuart RW Lefkowitz DL Starnes JD Margolin S Lefkowitz SS Inhibition of tumor necrosis factor and subsequent endotoxin shock by pirfenidone Int J Immunopharmacol 1998 20 12 685 695 10.1016/S0192-0561(98)00042-3 9877280

[B37] Asselah T Rubbia-Brandt L Marcellin P Negro F Steatosis in chronic hepatitis C: why does it really matter? Gut 2006 55 123 130 10.1136/gut.2005.069757 16344578 PMC1856395

[B38] Fartoux L Chazouillères O Wendum D Poupon R Serfaty L Impact of steatosis on progression of fibrosis in patients with mild hepatitis C Hepatology 2005 41 82 87 10.1002/hep.20519 15690484

[B39] Toyoda M Kitaoka A Machida K Nishinakagawa T Yada R Kohjima M Kato M Koton K Sakamoto N Shiota G Nakamuta M Nakashima M Enjoji M Association between lipid accumulation and the cannabinoid system in Huh7 cells expressing HCV genes Int J Mol Med 2011 27 5 619 624 21331443 10.3892/ijmm.2011.622

[B40] De Gottardi A Spahr L Ravier-Dall’Antonia F Hadengue A Cannabinoid receptor 1 and 2 agonists increase lipid accumulation in hepatocytes Liver Int 2010 30 10 1482 1489 10.1111/j.1478-3231.2010.02298.x 20602678

[B41] Westerbacka J Kotronen A Fielding BA Wahren J Hodson L Pertila J Perttilä J Seppänen-Laakso T Suortti T Arola J Hultcrantz R Castillo S Olkkonen VM Frayn KN Orešič M Yki-Järvinen H Splanchnic balance of free fatty acids, endocannabinoids, and lipids in subjects with nonalcoholic fatty liver disease Gastroenterology 2010 139 6 1961 1971 10.1053/j.gastro.2010.06.064 20600015

[B42] Coppola N Zampino R Bellini G Macera M Marrone A Pisaturo M Boemio A Nobili B Pasquale G Maione S Adinolfi LE Perrone L Sagnelli E Miraglia Del Giudice E Rossi F Association between a polymorphism in cannabinoid receptor 2 and severe necroinflammation in patients with chronic hepatitis C Clin Gastroenterol Hepatol 2014 12 334 340 10.1016/j.cgh.2013.05.008 23707465

[B43] Mendez-Sanchez N Zamora-Valdés D Pichardo-Bahena R Barredo-Prieto B Ponciano-Rodriguez G Bermejo-Martínez L Chavez-Tapia NC Baptista-González HA Uribe M Endocannabinoid receptor CB2 in nonalcoholic fatty liver disease Liver Int 2007 27 2 215 219 10.1111/j.1478-3231.2006.01401.x 17311616

